# Development of algorithms for the diagnosis and management of acute allergy in primary practice

**DOI:** 10.1016/j.waojou.2019.100022

**Published:** 2019-03-25

**Authors:** Pascal Demoly, Habib Chabane, Jean-François Fontaine, Delphine de Boissieu, Dermot Ryan, Elizabeth Angier, Jocelyne Just

**Affiliations:** Division of Allergy, Department of Pulmonology, Hôpital Arnaud de Villeneuve, University Hospital of Montpellier, Montpellier, France; INSERM UMRS 1136, Equipe – EPAR – IPLESP, Sorbonne Université, Paris, France; Department of Pediatrics, Hôpital Delafontaine, Saint Denis, France; Service des Maladies Respiratoires et Allergiques, Centre Hospitalier Universitaire, 45 rue Cognacq Jay, 51092, Reims Cedex, France; Service de Pneumologie et Allergologie Pédiatriques, Hôpital Universitaire Necker-Enfants Malades, Paris, France; Allergy and Respiratory Research Group, Usher Institute of Population Health Sciences and Informatics, University of Edinburgh, Teviot Place, Edinburgh, EH8 9AG, UK; Department of Clinical Immunology and Allergy, Northern General Hospital, Sheffield Teaching Hospital, Herries Road, Sheffield S5 7AU, UK; INSERM UMRS 1136, Equipe – EPAR – IPLESP, Sorbonne Université, Paris, France; Allergology Department, Centre de l'Asthme et des Allergies, Hôpital d'Enfants Armand-Trousseau – APHP, 26, Paris Cedex 12, France

**Keywords:** Allergy, Primary care, Diagnosis, AMSS, Allergy management support system, EAACI, European Academy of Allergy and Clinical Immunology, GP, General practitioner, NICE, National Institute for Health and Care Excellence

## Abstract

Most patients presenting with allergies are first seen in the primary care setting. However, inadequacies in training and available guidance for general practitioners (GP) have been identified as significantly impacting the quality of care for these patients, resulting in inefficient use of healthcare resources. To address the lack of available guidance, a working group of French allergists has developed a series of online tools aimed at GPs. The expert panel developed algorithms for the diagnosis and treatment of common allergies by incorporating deliberations based on clinical guidelines and experience. In addition, they developed tables of common symptoms and detailed clinical cases that guide GPs through the typical decisions they are faced with in line with current best practice. These tools translate evidence-based recommendations from international clinical guidelines, outlining the key steps involved and assisting the physician in making decisions at each step. In addition to targeting improvements in diagnosis and standard of primary care, the tools also aim to reduce the burden on specialist allergy services by enabling GPs to diagnose and treat mild and moderate allergies, referring only severe and/or atypical cases to secondary care. The tools are adapted to the high primary care workload, enabling the physician to access essential information rapidly without unnecessary referrals to specialist allergy services.

## Introduction

### A central role for GPs in allergy management

The majority of patients seeking medical advice for allergies are first seen in the primary care setting.^1^, ^2^ It is estimated that allergic diseases account for around 8% of primary care consultations in the United Kingdom (UK).^1^ The number of people with allergic diseases such as asthma continues to grow; in the United States, about 20 million people had asthma in 2001, compared with about 25 million in 2009 (or 8% of the population).^3^ As a result, primary care physicians are increasingly expected to diagnose and manage mild and moderate allergies, referring only more complex or severe cases to specialist services.^2^ Nevertheless, many mild to moderate patients are referred to specialist allergy services because general practitioners (GPs) lack confidence in diagnosing and managing allergic diseases.^4^ Although GPs have usually received some form of training in allergy, many often feel ill equipped for this task, owing to lack of specialized training, difficulties in accessing specialists, lack of expertise and facilities for investigating allergic conditions and managing specific areas such as food allergy.^1^, ^4^, ^5^, ^6^ In addition to increasing prevalence, it has been reported that the diagnosis and management of allergic patients in primary care is becoming difficult due to the increasing complexity of allergic diseases.^7^

### A need for good allergy management in primary care

Inappropriate management of allergies and asthma causes a considerable financial burden and negatively impacts the quality of life for millions of people with allergic disease.^8^, ^9^, ^10^, ^11^, ^12^ Inadequacies in primary care allergy services have been identified as having a significant impact on the quality of care for allergic patients.^1^, ^13^ There is evidence that allergies are under-diagnosed and often incorrectly treated in the primary care setting^14^; for example, inadequate care pathways result in poor referral practices and delays in patient management, resulting in poor patient outcomes and a waste of healthcare resources.^1^ In 2004, a UK Commons Health Committee report cited lack of allergy knowledge among primary care physicians as a principal cause of distress to patients.^13^ This finding was supported by studies that found allergy training and information available to primary care physicians was inadequate^2^, ^4^, ^5^, ^6^, ^15^; physicians did not receive structured allergy instruction during their training, very few were familiar with guidelines for the management of allergic diseases, and continuing medical education programs were found to be inadequate, according to a systematic review of pathways for the delivery of allergy services.^1^ Specifically, physicians lacked education in recognizing allergic diseases, diagnostic workups, and referrals.^2^ Less than 30% felt comfortable interpreting laboratory tests for food allergy or felt adequately prepared to care for children with food allergies,^16^ despite up to 8% of children in the United States being affected by hypersensitivity reactions to various foods.^17^

In addition, more than three-quarters of GPs felt they had inadequate knowledge of allergen immunotherapy, and were not confident referring patients for specialist care.^15^ As the standard of allergy care in the primary care setting strongly influences allergy prevention and management, and ultimately patient quality of life and satisfaction,^2^ it is important that GPs have adequate training and access to quality medical advice. Although continuous medical education is needed for all doctors, many have busy agendas, and need to close their practice when physical presence for training is required. In this context, online tools, such as webinars, on-demand videos, and decision-making tools may be a valuable solution.

### Online tool development for allergy management in primary care

The object of this study was therefore to develop online tools aimed at providing practical advice and guidance to GPs confronted with a patient showing possible symptoms of allergy. In addition to helping with diagnosis and management, the aim was to also to assist GPs in determining when referral to a specialist is needed.

## Methods

### Content development

The expert group comprised of two allergists with long postgraduate training experience in general practice, two pediatricians, one dermatologist, and one respiratory physician, who met three times a year over a period of 5 years. The group searched for published needs-based assessments and guidance from professional societies and expert groups designed to help primary care physicians to diagnose and manage allergic patients.

Based on identified needs and clinical guidelines, the expert group developed structured algorithms for patients presenting with symptoms suggestive of allergic presentations commonly encountered in primary care, such as allergic respiratory symptoms, acute allergic reactions to food or drugs, and other typical allergic symptoms. Particular attention was placed on danger signs and situations that must lead to referral to an allergist and on the precautions to take while waiting for the visit. Algorithms were designed to leave the GP as much freedom as necessary, whilst taking into account the high prevalence of allergies (there are insufficient allergy specialists in the world to handle all patients), the general experience of GPs, and the lack of knowledge they may have in the fast evolving world of allergy. The tools were reviewed by the expert group for clarity and ease of use prior to being posted for real-life clinical practice use. In addition, a table of the most common allergy symptoms and a series of clinical examples were developed.

### GP review

Following development and review by the experts, all materials were reviewed by a group of five GPs with interest in allergic disease prior to being made available for general use. The review panel met on several occasions to discuss modifications and used an informal Delphi process to reach a consensus. Modifications to the draft algorithms were made once 100% agreement was reached. The algorithms were then made directly and freely available online for crowdsourcing use at http://www.diagnosticallergie.fr. No systematic attempt to record uptake, usefulness, and acceptance of the tools by the GP community was made.

## Results

### Identification of unmet needs

Using a modified Delphi technique, a group of Swedish allergy experts identified core competencies necessary for the management of allergic patients in primary care.^18^ They found that these should include diagnosis and treatment of anaphylaxis and interpretation of laboratory data. Primary care physicians should be able to investigate, treat and control asthma and allergic reactions to food and drugs. Finally, they should have an understanding of provocation testing and how it is assessed.^18^ The most significant finding was that primary care physicians should be able to communicate with allergic patients about their disease and to exercise necessary caution when dealing with pediatric patients.^18^ The experts identified a number of primary care guidelines, including those from the UK National Institute for Health and Care Excellence (NICE); however, the majority of these focused on specific allergies, such as food allergy.^19^, ^20^, ^21^, ^22^

### Tools

**Fig. 1:**
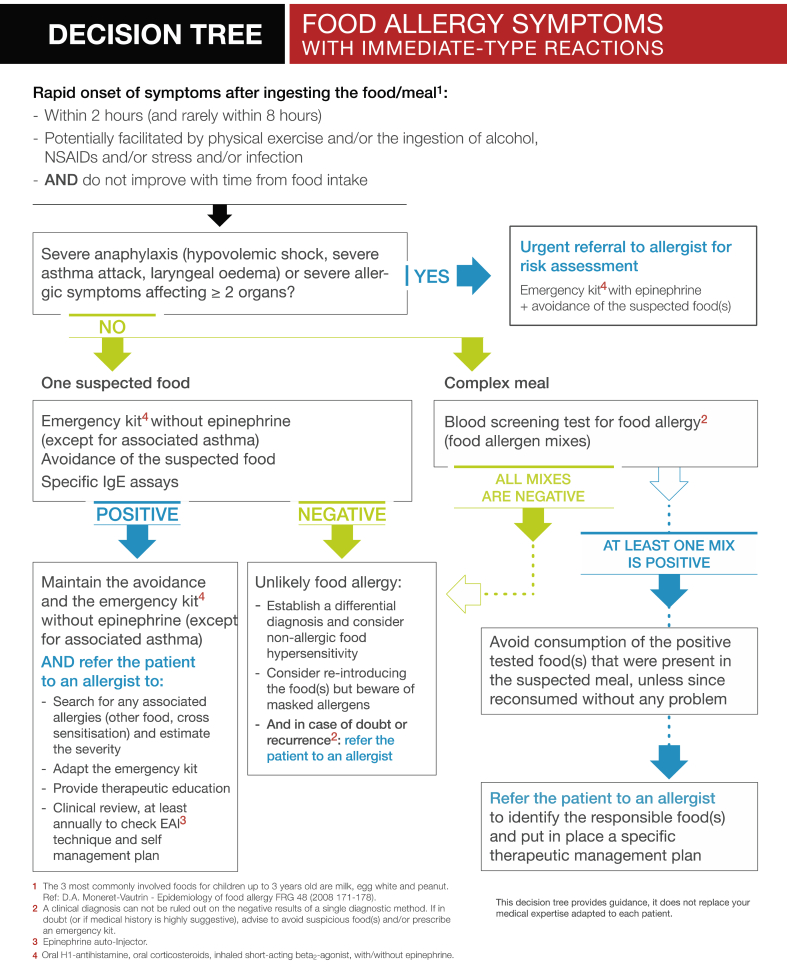

**Fig. 2:**
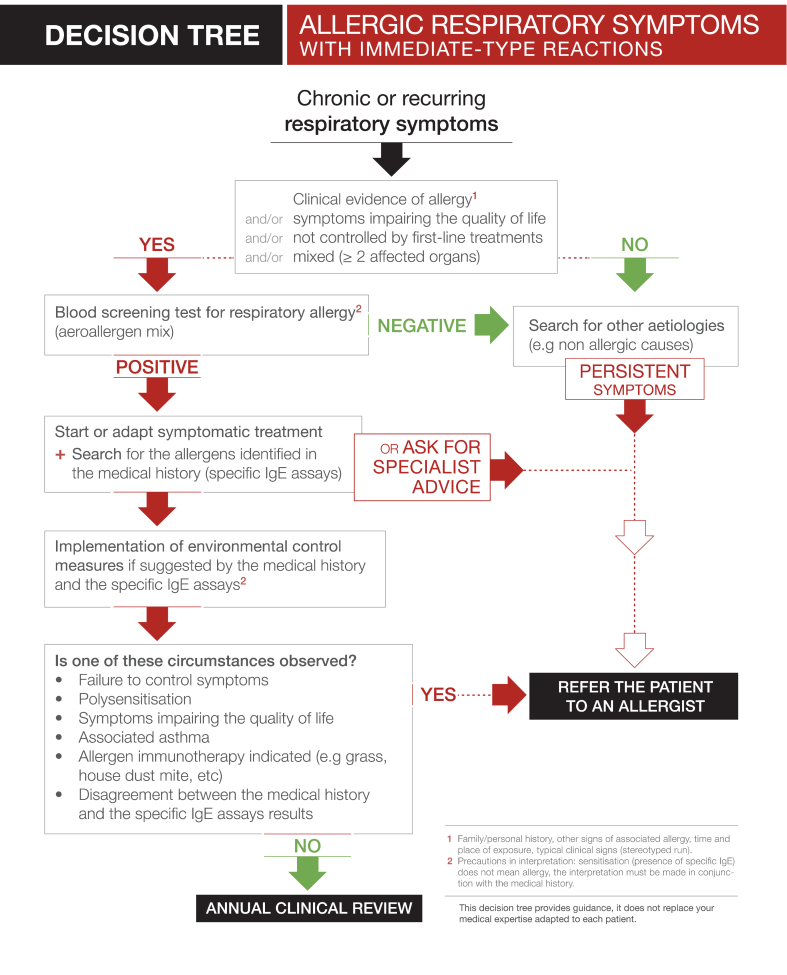

**Fig. 3:**
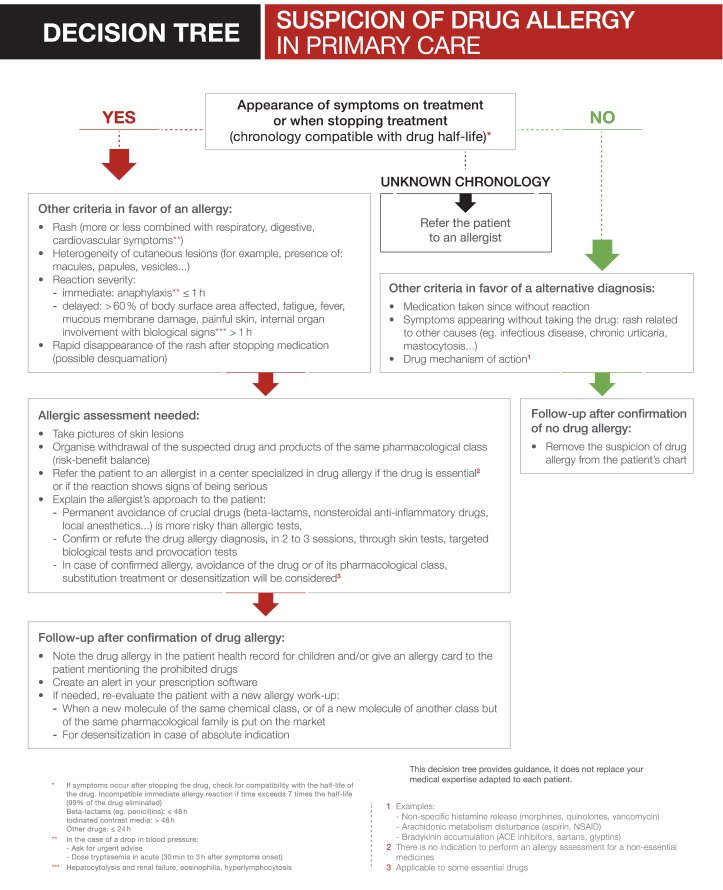

The algorithms developed by the expert group are presented in Fig. 1, Fig. 2, Fig. 3. They translate evidence-based recommendations from clinical guidelines into tools that outline the key steps and assist the physician in making decisions at each step for patients with symptoms suggestive of food allergy, allergic respiratory symptoms, or suspected drug allergy. The decision trees list common allergens, and provide step-by-step guidance on obtaining clinical history and identifying target allergens, when to use laboratory testing and choice of test, interpretation of test results, allergen avoidance, treatment options, and when to obtain specialist advice or refer the patient to secondary services. In the case of food allergy (Fig. 1), care was taken to stress that even an isolated event of consuming an allergenic foodstuff could result in a serious allergic reaction. Each step is accompanied by an explanation to improve clinical reasoning skills.

The algorithms were based on detailed cases developed by the experts that were designed to represent common, everyday situations, and guide the physician through the typical decisions they are faced with, in line with current best practice. To ensure coherence, care was taken to make sure the guidance was in line with that of institutional recommendations.

**Fig. 4:**
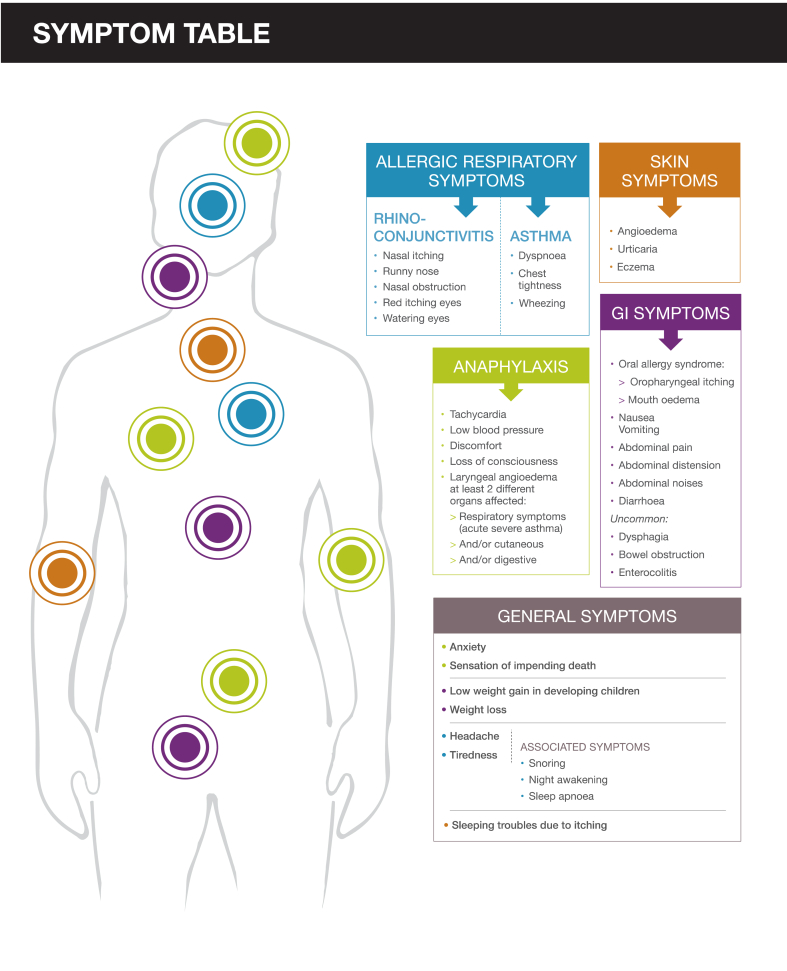

The group also developed a table of the most common symptoms seen in cutaneous, respiratory and food allergies, and anaphylaxis, as well as general symptoms common to all allergy presentations (Fig. 4). In addition, a series of clinical examples covering recurring pediatric rhinitis, evolution of adolescent rhinitis into asthma, pollen allergy presenting with complex symptoms, and non-allergic rhino-sinusitis, were developed, along with a series of two-minute videos on the choice of laboratory tests, diagnosis of asthma, interpreting the results of specific-IgE tests, and how to obtain reimbursement for laboratory tests, which are available free of charge from http://www.diagnosticallergie.fr.

## Discussion

In this study, we describe the development of tools designed to assist primary care physicians manage patients with symptoms suggestive of an acute allergic reaction. The tools were designed to be easily accessible and intuitive, and were developed with the time restrictions of a high primary care workload in mind. Content is consistent with recommendations from evidence-based clinical guidelines and with unmet needs identified by the expert group and in published surveys. The online route of dissemination is aligned with the outcome of an educational needs survey that reported that online guidelines were the preferred learning modality for primary care physicians.^15^ No subscription or registration is required for access, making them easily available to all GPs.

Since the project was initiated, other guided pathways designed to assist GPs diagnose and treat allergic patients have been published, underlining the considerable unmet need for simplified tools adapted to primary practice identified by the expert group and other authors. As a means to enable GPs to care for patients with mild and moderate allergies, general guidelines from the European Academy of Allergy and Clinical Immunology (EAACI) proposed simplified pathways for recognition and diagnosis of common allergic diseases that can be adapted to local practices.^20^ These guide the physician in obtaining clinical history, *in vivo* or *in vitro* testing and the need for additional procedures, such as challenge tests, to establish a diagnosis, but do not provide guidance on management.^20^ In 2017, a group of Dutch allergists, dermatologists, primary care physicians and allergy researchers, developed an allergy management support system (AMSS) to support GPs with the diagnosis and management of allergic patients.^7^, ^23^ Depending on the answers to a disease history questionnaire completed by the patient, and specific-IgE test outcomes, the physician is guided on diagnostic classification and severity, and receives management recommendations based on primary care and specialists' allergy guidelines complemented with clinical knowledge and expert opinion. The AMSS was validated against an assessment by an allergy specialist, which was regarded as the gold standard.

Unlike the AMSS, our tools did not undergo a full validation process, as this was deemed too costly and would have delayed availability to the GP community; therefore, the tools were made directly available online for crowdsourcing use at http://www.diagnosticallergie.fr. While this could be seen as a limitation, our systematic development process was similar to that of the AMSS, which demonstrates that using this approach it is possible to develop a validated algorithm of diagnostic and management recommendations for GPs who encounter allergic patients.^7^

In 2018–2019, three of the experts together with four European GP/allergists are reviewing EAACI questionnaires, national guidelines, original articles, and personal documents as a basis for updating and revising the algorithms. The group will also review the results of questionnaires on dermatitis, urticaria and anaphylaxis from the GP community. There are plans to expand the tools to include other commonly encountered allergic diseases, such as contact allergy, allergy to hymenoptera and other arthropod venoms, polysensitization, and less common allergic diseases, as well as webinars detailing how the tools can be used in the GP consultation, with simulated cases and responses by GPs, and commentary from patients and specialists. The group will work on investigating validity, feasibility, and uptake of the tools.

In 2013, the EAACI Task Force for Allergy Management in Primary Care found that the current model, in which the care for allergic patients is predominantly specialist-based, was not sustainable with increasing disease prevalence.^14^ There is evidence to indicate that increasing awareness of common allergic conditions among primary care physicians could significantly reduce the burden on specialist allergy services.^24^ Studies in the UK and Ireland that reviewed primary care referrals to allergy clinics observed that only 9%–23% of patients referred to specialists were diagnosed as having an allergy, with the majority of other referrals consisting of patients with other symptoms (e.g. chronic spontaneous urticaria, non-allergic food hypersensitivity, or non-specific symptomatology).^24^, ^25^ It was also estimated that up to half of allergy referrals in the UK could have been easily managed in primary care, had the physicians been trained appropriately.^25^, ^26^

In addition to targeting improvements in diagnosis and standard of care, the tools described here also aim to improve the use of healthcare resources, by helping the physician to take an allergy-focused clinical history, choose the most appropriate laboratory tests and interpret them within the context of the clinical history. They provide clear guidance and signposting on when to refer the patient for specialist care, which could lead to operational cost savings. The online resources are adapted to the high workload of the primary care physician, and enable easy and rapid access to essential information without unnecessary referrals to specialist allergy services, which will improve the patient experience by having their healthcare needs met sooner and closer to home. This could achieve the triple aim of better quality healthcare, increased value, and a better patient experience.

## Declarations

### Ethics approval and consent to participate

Not applicable.

### Consent for publication

Not applicable.

### Availability of data and material

Data sharing is not applicable to this article as no datasets were generated or analyzed during the current study.

### Competing interests

The authors declare that they have no competing interests.

### Funding

The project was supported by an educational grant from Thermo Fisher Scientific. The funding body was not involved in designing the materials or writing the manuscript.

### Authors' contributions

Pascal Demoly contributed to the original algorithm idea and construction of the document; together with Habib Chabane, Jean-François Fontaine, Jocelyne Just, and Delphine de Boissieu, they built the algorithms and contributed to writing the manuscript. Dermot Ryan and Elisabeth Angier revised the original materials. All authors contributed to fine-tuning the algorithms and to revising the manuscript.
